# Combined topical and systemic administration with human adipose-derived mesenchymal stem cells (hADSC) and hADSC-derived exosomes markedly promoted cutaneous wound healing and regeneration

**DOI:** 10.1186/s13287-021-02287-9

**Published:** 2021-05-01

**Authors:** Yang Zhou, Bo Zhao, Xin-Liao Zhang, Yi-jun Lu, Shou-Tao Lu, Jian Cheng, Yu Fu, Lin Lin, Ning-Yan Zhang, Pei-Xin Li, Jing Zhang, Jun Zhang

**Affiliations:** 1 Research Center for Translational Medicine, Shanghai East Hospital, School of Medicine, Tongji University, Shanghai, 200092 China; 2 Key Laboratory of Spine and Spinal Cord Injury Repair and Regeneration of Ministry of Education, Stem Cell Translational Research Center of Tongji Hospital, School of Life Science and Technology, Tongji University, 389 Xincun Road, Shanghai, 200065 China; 3 Shanghai Institute of Stem Cell Research and Clinical Translation, Shanghai, China; 4 Tongji Lifeng Institute of Regenerative Medicine, Tongji University, Shanghai, China

**Keywords:** Adipose mesenchymal stem cells, Exosome, Wound healing

## Abstract

**Background:**

Cutaneous wound healing and regeneration have become a recognized health challenge in the world, which causes severe damage to the mental and physical health of patients. Human adipose-derived mesenchymal stem cells (hADSC) play an essential role in wound healing via their paracrine function. Exosomes secreted by hADSC may contribute to this progress. In this study, we investigated the potential clinical application roles of hADSC and hADSC-derived exosomes (hADSC-Exo) in cutaneous wound healing.

**Methods:**

hADSC-Exo was isolated from human hADSC by ultracentrifugation. Mice were subjected to a full-thickness skin biopsy experiment and treated with either control vehicle or hADSC or hADSC-Exo by smearing administration (sm) or subcutaneous administration (sc) or intravenous administration (iv). The efficacy of hADSC and hADSC-Exo on wound healing was evaluated by measuring wound closure rates, histological analysis.

**Results:**

Combined application of local hADSC-Exo smearing and hADSC/hADSC-Exo intravenous administration offered the additional benefit of promoting wound healing, accelerating re-epithelialization, reducing scar widths, and enhancing angiogenesis and collagen synthesis. Either topical application of hADSC-Exo or systemic administration with hADSC/hADSC-Exo appeared more effective in stimulating cell proliferation, inhibiting cell apoptosis and inflammation, and promoting skin elasticity and barrier integrity, with increased genes expression of PCNA, VEGF, collagen III, Filaggrin, Loricrin, and AQP3, with decreased genes expression of TNF-alpha.

**Conclusion:**

Our findings suggest that the combined administration of hADSC/hADSC-Exo can facilitate cutaneous wound healing and reduce scar formation. These data provide the first evidence for the feasibility of smearing of hADSC-Exo as a cell-free therapy in treating cutaneous wounds, and the potential clinical value of combined administration of hADSC/hADSC-Exo.

**Supplementary Information:**

The online version contains supplementary material available at 10.1186/s13287-021-02287-9.

## Background

Cutaneous wound healing is an intricate and highly orchestrated physiological process, including hemostasis, inflammation, tissue formation, and tissue remodeling [1–3]. The normal wound healing process is disrupted and prolonged in some disease conditions resulting in chronic non-healing wounds, for instance, diabetic ulcers or pathological scars, e.g., keloid scars [2, 4, 5]. This has caused severe damage to the patients’ economic situation, as well as their mental and physical health. Thus, it is urgent for us to find an effective way to shorten the wound’s healing time and reduce scar formation. Previous studies have shown that hADSC can enhance wound healing, inhibit scar formation, and resist photoaging through a paracrine mechanism [6–11]. Recently, multiple studies have documented that exosomes play a crucial role in paracrine mechanisms. Exosomes have received much attention in basic research and clinical utility [12, 13].

Exosomes, 30–200 nm vesicles originate from the rough endoplasmic reticulum, encapsulate various active substances [14, 15]. Exosomes are significant carriers of signals between cells. They play vital roles in intercellular communication via transferring specific DNAs, proteins, microRNAs, lipids, mRNAs, and other signaling molecules to target cells [15–17]. Exosomes are a vital mediator of paracrine function, found in many body fluids, including the saliva, serum, and urine. A growing body of evidence has demonstrated that MSC-derived exosomes’ therapeutic potential for tissue responses to injury, infection, and disease [15, 18–21]. Research documents that exosomes show similar capacities to host cells and exhibit no apparent side effects, e.g., malignant transformation, vascular obstructive risks, and immunogenicity, demonstrating that therapy based on exosomes is considerably safe and promising for tissue regeneration therapy compared to the direct utilization of cells [22–26].

Herein, we used a mouse model to explore the effects of hADSC and hADSC-Exo on repairing wound skin. We found that the topical medication of smearing hADSC-Exo is more beneficial for promoting skin damage repair than the subcutaneous injection of hADSC or hADSC-Exo. This is mainly because hADSC-Exo can promote cell proliferation, angiogenesis, collagen synthesis, and skin barrier function repair, which benefits wound healing. For systemic delivery, local administration combined therapy with the intravenous injection of hADSC and hADSC-Exo offered additional benefit over either therapy alone to enhance cutaneous regeneration. Herein, we, for the first time, access the therapeutic effect of hADSC and hADSC-Exo for wound healing and provide simple and effective clinical treatment suggestions for acute and chronic wounds.

## Methods and materials

### Isolation and identification of human adipose mesenchymal stem cells (hADSC)

Human adipose tissue samples were obtained from a healthy female donor with the donor’s written informed consent as approved by the East Hospital Affiliated to Tongji University. This healthy female donor is 30–35 years of age with HIV-negative, HBV-negative, HCV-negative, CMV-negative, EBV-negative, and TP-negative. hADSC were isolated from adipose tissue as previously reported, and they were positive for CD73, CD90, and CD105, and negative for CD45 and HLA-DR [22]. In brief, adipose tissues were washed 2–3 times with sterile normal saline and then centrifuged at 1500 rpm for 10 min. After centrifugation, the liquid was divided into three layers. The upper layer of floating adipocytes was treated with collagenase type I for 30 min at 37 °C with intermittent shaking. The cell pellets were separated by centrifugation at 1500 rpm for 10 min and then suspended in complete medium containing Minimum Essential Medium α (MEM) (Gibco,USA) and 10% UltraGRO™-Advanced (Helios Bioscience, USA) supplemented with 100 U/mL penicillin-streptomycin (Gibco, USA). The primary hADSC were cultured for 4–5 days until they reached confluence (defined as passage 0). The cells were then harvested by 0.25% trypsin-EDTA (Invitrogen, USA) and diluted 1:2 to 1:3 and plated for subculture, and the cell used in experiments during the third-seventh passages. After 3–7 passages, the hADSC were harvested and stained with fluorescence-conjugated antibodies (BD Biosciences): anti-CD73-PE, anti-CD105-BV421, anti-CD90-APC, anti-CD45-FITC, and anti-HLA-DR-PE-Cy5-conjugated. Isotype control IgG was used to stain the cells as a control. After two washes with PBS, the cells were analyzed by using a flow cytometer (BD FACSVerse™, Becton-Dickinson, San Jose, CA, USA), and the data analysis was conducted using the Flowjo software (Tree Star Inc., USA).

For the adipogenic, osteogenic, and chondrogenic differentiation, hADSC were incubated into adipogenic, osteogenic, and chondrogenic differentiation medium (Cyagen, China) for 3 weeks. For adipogenesis, cell pellets were stained with oil red O to detect the intracellular accumulation of lipid vacuoles. Osteogenesis was demonstrated by the formation of bone matrix assessed by Alizarin Red S staining. Chondrogenesis was confirmed by the secretion of sulfated glycosaminoglycans stained with Alcian Blue.

### Isolation and identification of hADSC-Exo

Exosomes were isolated from culture supernatant of hADSC as demonstrated previously [25]. Briefly, the culture media of confluent hADSC was collected and centrifuged at 300×*g* for 10 min to remove cells. Transfer the supernatant from the centrifugal tube to the new centrifugal tube and centrifuge at 2000×*g* for 10 min. To remove cell debris, transfer the supernatant to the new centrifugal tube again and centrifuge at 10,000×g for 30 min. After filtered through a 0.2-μm filter, the supernatant was again collected and centrifuged at 100,000×*g* for 2 h at 4 °C. The supernatant was then discarded because the pellet now contained exosomes. Exosomes were resuspended in 1 × PBS and stored at − 80 °C for the following experiments. Exosomes were quantified with the total exosome protein assay. The quantification of the total exosome protein was addressed in the methods of Western Blot. Generally, 100μg exosomes could be isolated from 20 ml culture medium of 3–7 passages 10^× 9^ hADSC. The morphology of exosomes was observed under a transmission electron microscope (TEM, FEI, USA). The exosomes were diluted with 1× PBS (10-fold), filtered the mixture into a new centrifuge tube with a 0.22 μm filter, and then subjected to Nanosight tracking analysis (NTA, Malvern, USA).

### In vivo wound healing experiments in an animal model

All procedures were approved by the Animal Research Committee of Tongji University. Male ICR mice (7 weeks old, weighing 28–35 g) maintained under specific pathogen-free (SPF) conditions, which were purchased from Shanghai SLAC Laboratory Animal Co., Ltd., were used in this study. After shaving the mice, we created a 1.5 × 1.5 cm full-thickness wound on the back of mice to confirm the effects of hADSC and hADSC-Exos on cutaneous wound healing. Mice were randomly divided into 5 different groups: untreated group (control group), rhEGF local smearing group (positive group), 1 × 10^6^ hADSC in 200 μl PBS subcutaneous administration (sc) group (hADSC-sc group), 200 μg exosome in 200 μl PBS subcutaneous administration group (hADSC-Exos-sc group), and 200 μ g exosome in 200 μl PBS local smearing group (hADSC-Exos-sm group). The mice were housed individually and were treated with above these measures once a day. The wound areas were measured at days 0, 3, 7, 10, and 12 and calculated with image analysis software. Mice were killed at day 12 after surgery. Half of the wound skin tissues were collected for immunohistochemistry staining and western blot analysis.

To further confirm the relationship between different frequencies and the optimal protocol for hADSC-Exo-sm, we divided the mice into three groups and hADSC-Exo-sm was carried out by three different frequency of administration for up to 12 days of continuous administration as follows: Qd: once a day, Bid: twice a day, and Tid: three times a day. A single application dose of hADSC-Exo-sm is 200 μ g in 200 μl PBS. The wound areas were measured at days 0, 3, 7, 10, and 12 and calculated with image analysis software. Wound skin tissues were collected on day 12 following treatment for immunohistochemistry staining. To further compare the effects of topical hADSC-Exo-sm treatment and systemic medication, hADSC, hADSC-Exo, and PBS were injected by tail vein injection 1 day after wound modeling. Similarly, the wound tissues were collected and stained for immunohistochemical analysis.

### Histological analysis

The skin at the wound site of each group of mice was cut and fixed. For the histological evaluation, the worst part of each wound healing condition was selected for analysis. Tissue sections were fixed overnight in 10% buffered formalin at room temperature. Next, the samples were transferred to 70% ethanol for an additional 48 h and then embedded in paraffin. Serial sections from the middle of the wound were made in a 5-μm cross-section.

The sections were stained with hematoxylin and eosin H&E and Masson’s trichrome. Immunohistochemistry was performed as described above. The dewaxed sections were washed in PBS, and the endogenous peroxidase activity was quenched by immersion in 2%(v/v) hydrogen peroxide for 5 min. The antigen retrieval was repaired by incubation with sodium citrate buffer for 30 min. After washing in PBS, the sections were sealed with 1.5% goat serum at room temperature for 30 min and then incubated with primary antibodies provided in Table 1, overnight at 4 °C, and then washed with PBS and incubated with respective secondary antibody (goat anti-rabbit or goat anti-mouse, ZSGB-BIO, China) for 20 min at room temperature. Immunohistochemical staining was developed using the DAB substrate system (DAKO, Denmark). Images were collected using a BX53 microscope (Olympus, Japan).

**Table 1:** The dilutions of the primary antibodies used for IHC

Protein name	Dilution	Company	Area
Collagen I	1:1000	Abcam	Cambridge, UK
Collagen III	1:2000	Abcam	Cambridge, UK
Ki67	1:400	CST	MA, USA
PCNA	1:1000	Boster	CA, USA
Cleaved-Caspase 3	1:100	Abcam	Cambridge, UK
VEGF	1:500	Abcam	Cambridge, UK
CD31	1:1000	Abcam	Cambridge, UK
Filaggrin	1:500	Abcam	Cambridge, UK
Loricrin	1:200	Abcam	Cambridge, UK
AQP3	1:500	Abcam	Cambridge, UK
TNF-α	1:200	Boster	CA, USA
CD14	1:100	Abcam	Cambridge, UK
CD19	1:200	ZSGB-BIO	Beijing, China
CD68	1:200	Boster	CA, USA

IHC image analysis was performed using Image-Pro Plus 6.0 software. Three animals per group were analyzed for immunohistochemical staining. For each sample, at least 5 fields (× 400 magnification) were randomly selected for analysis. For CD14, VEGF, TNF-α, PCNA, Ki67, IL-6, CD68, CD19, and Cleaved Caspase3 analyses, data are represented as the percentage of positive cell numbers divided by total cell numbers. For collagenous fiber in Masson, collagen I, collagen III, AQP3, and Loricrin burden analyses, data are reported as the mean density (IOD/Area) of positively stained regions. The average number of CD31-positive small vessels were manually counted in five random fields. All histological assessments were performed by two independent observers.

### Western blotting

Exosomes were lysed with RIPA lysis buffer (ThermoFisher, USA) supplemented with the protease inhibitor cocktail and phosphatase inhibitor mini-tablets (Pierce, USA). The lysate was centrifuged at 3000 rpm for 15 min at 4 °C, and the supernatant was quantitated using the Micro Bicinchoninic Acid (BCA) Protein Assay Kit (Pierce). The total 40μg protein was resolved by SDS-PAGE on 10% polyacrylamide gels and was transferred onto Hybond-P polyvinylidene difluoride (PVDF) membrane (Millipore). The membranes were incubated with antibodies overnight at 4 °C and further incubated with the appropriate horseradish peroxidase (HRP)-conjugated secondary antibodies (Abcam, Cambridge, UK) for 1 h at room temperature. The blots were visualized by enhanced chemiluminescence (ECL) detection reagents (Bio-Rad, USA). Images were documented, and band density was analyzed by Amersham Imager 600 (GE). The antibodies were used in this study as follows: anti-CD9 (Abcam, Cambridge, UK) and anti-CD63 (Abcam, Cambridge, UK).

### Statistical analysis

Statistical analysis. Statistical analysis was performed using Graphpad Prism software. Two-tailed Student’s *t* test was applied when there were only two groups of samples. In the case of more than two groups of samples, one-way ANOVA was used with one condition, and two-way ANOVA was used with more than two conditions. ANOVA analysis was followed by post hoc Bonferroni’s correction for multiple comparisons. *p* < 0.05 was taken as statistically significant; *p value < 0.05, **p value < 0.01, ***p value < 0.001, and ****p value < 0.0001. The data displayed a normal distribution. The estimated variance was similar between experimental groups. Data are presented as the mean ± SD or ± SEM, as indicated in the figure legends.

## Results and discussion

### Characterization of hADSC and hADSC-Exo

**Fig. 1:**
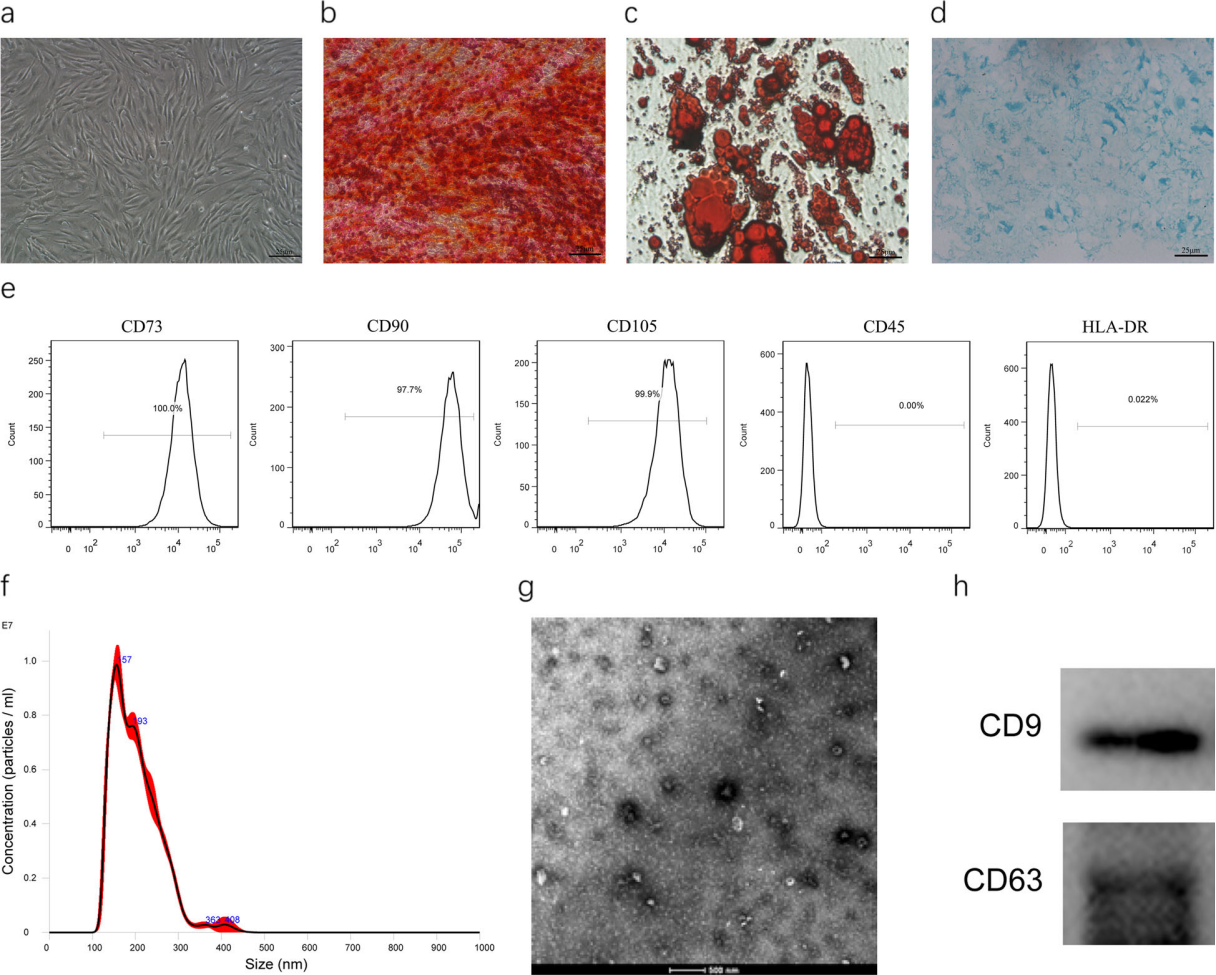
Isolation and characterization of hADSC and hADSC-Exo. **a** Spindle hADSC were observed through the microscope. Scale bar = 25 μm. **b**–**d** Adipogenic, osteogenic, and chondrogenic differentiation of hADSC. Cells differentiated into adipocyte, osteoblasts, and chondrogenic were detected using Oil Red O, Alizarin Red, and Alcian Blue, respectively. Scale bar = 25 μm. **e** Flow cytometry of hADSC surface markers CD29, CD73, CD105, SSEA-3, SSEA-4, and HLA-DR. **f** Nanoparticle analysis of ADSC-Exo. **g** TEM analysis of exosomes. Scale bars = 500 nm. **h** Immunoblotting for CD9 and CD63 in exosomes

hADSC cells were grown as described in the “Methods” section and had typical fibroblastic morphology as indicated in Fig. 1a. The cells exhibited adipogenic, osteogenic, and chrondrogenic differentiation properties as illustrated by the oil red O staining of the adipocytes, Alizarin Red S staining of the osteoblasts, and Alcian Blue staining of chrondrogenic, and respectively, as indicated in Fig. 1b–d. The flow cytometry data demonstrated that hADSC cells were remarkably positive for MSC surface biomarkers consisting of CD105, CD73, and CD 90, and negative for HLA-DR and CD45.

Exosomes were extracted from the supernatants of hADSC by ultra-spinning. The electron microscopy data demonstrated that hADSC-Exo had a saucer-shaped morphology, with diameters ranging between 30 to 100 nm (Fig. 1f), consistent with previously reported exosomes. Nanosight analysis was employed to explore the particle concentration, size distribution, and dynamic tracking, as indicated in Fig. 1g. The western blotting data revealed some exosome biomarkers, including CD9 and CD63, expressed in exosomes, further validating their identity as exosomes. Altogether, these data demonstrated that hADSC-Exo was isolated successfully and was consistent with defined exosomes.

### hADSC-Exo-sm promotes cutaneous wound healing

**Fig. 2:**
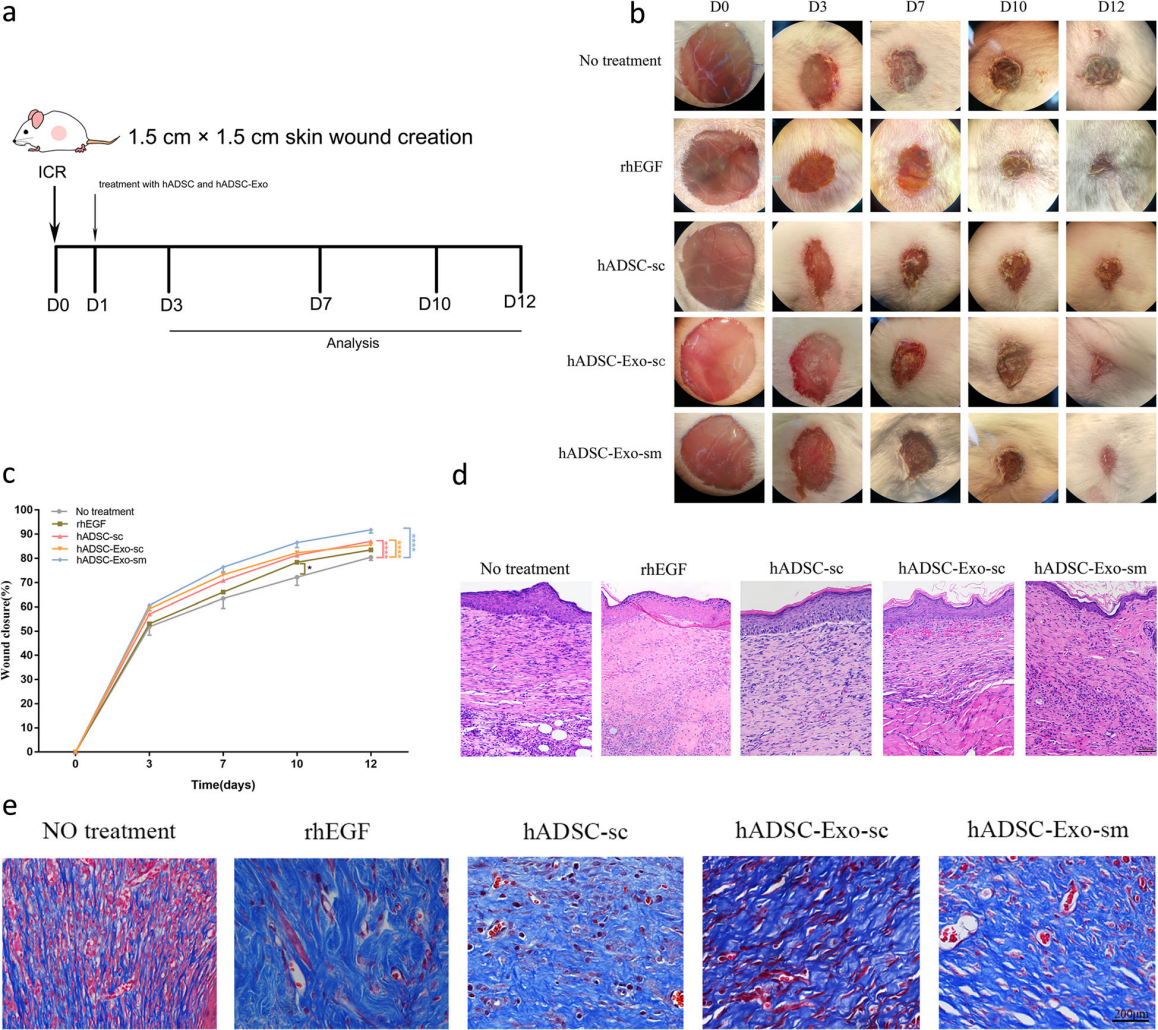
hADSC-Exo enhance cutaneous wound healing in an in vivo mouse model. **a** The study protocol. **b** Representative photographs of full-thickness excision wounds treated with rhEGF (positive control), hADSC, or hADSC-Exo. hADSC-Exo were administered either by sm or sc for 12 days. **c** Quantitative analysis of wound healing in each group. **d** H&E staining of wounded skin sections in different groups at day 12 post-wounding. Scale bar = 250 μm. **e** Masson staining of wounded skin sections in different groups at day 12 post-wounding. Results are presented as mean ± standard error of the mean; *n* = 8 for each group. *p < 0.05, **p < 0.01, ***p < 0.001, and ****p < 0.0001 vs vehicle control group. sm smearing administration, sc subcutaneous administration

To evaluate the effect of hADSC and hADSC-Exo on cutaneous wound healing, we apply the mice model. The full-thickness cutaneous wounds were created on the dorsal skin areas of male ICR mice, followed by the treatment of hADSC-sc, hADSC-Exo-sc, and hADSC-Exo-sm on the wound sites once a day for 14 days (Fig. 2a). As a positive control, Recombinant Human Epidermal Growth Factor Hydro Gel (1 g/cm, rhEGF, trade name YiFu) was locally smearing administered once daily. As in Fig. 2b, c, both subcutaneous administration and smearing administration of hADSC and hADSC-Exo significantly promote the cutaneous wounds closure rate compared to the controls. Notably, the hADSC-Exo-sm group and the rhEGF group appeared to have a significantly better velocity of the wound healing process than the other groups on days 7, 10, and 12 post wounding. Surprisingly, the better epithelial tissue was observed in the hADSC-Exo-sm groups in HE staining observation (Fig. 2d). Masson staining (Fig. 2e) of tissue sections also illustrated that wounds treated with hADSC-Exo-sm exhibited well-reorganized collagen fibers compared to other post-wounding wounds, implying that hADSC-Exo-sm treated wounds exhibited remarkably shorten healing time, as well as narrower scars.

**Fig. 3:**
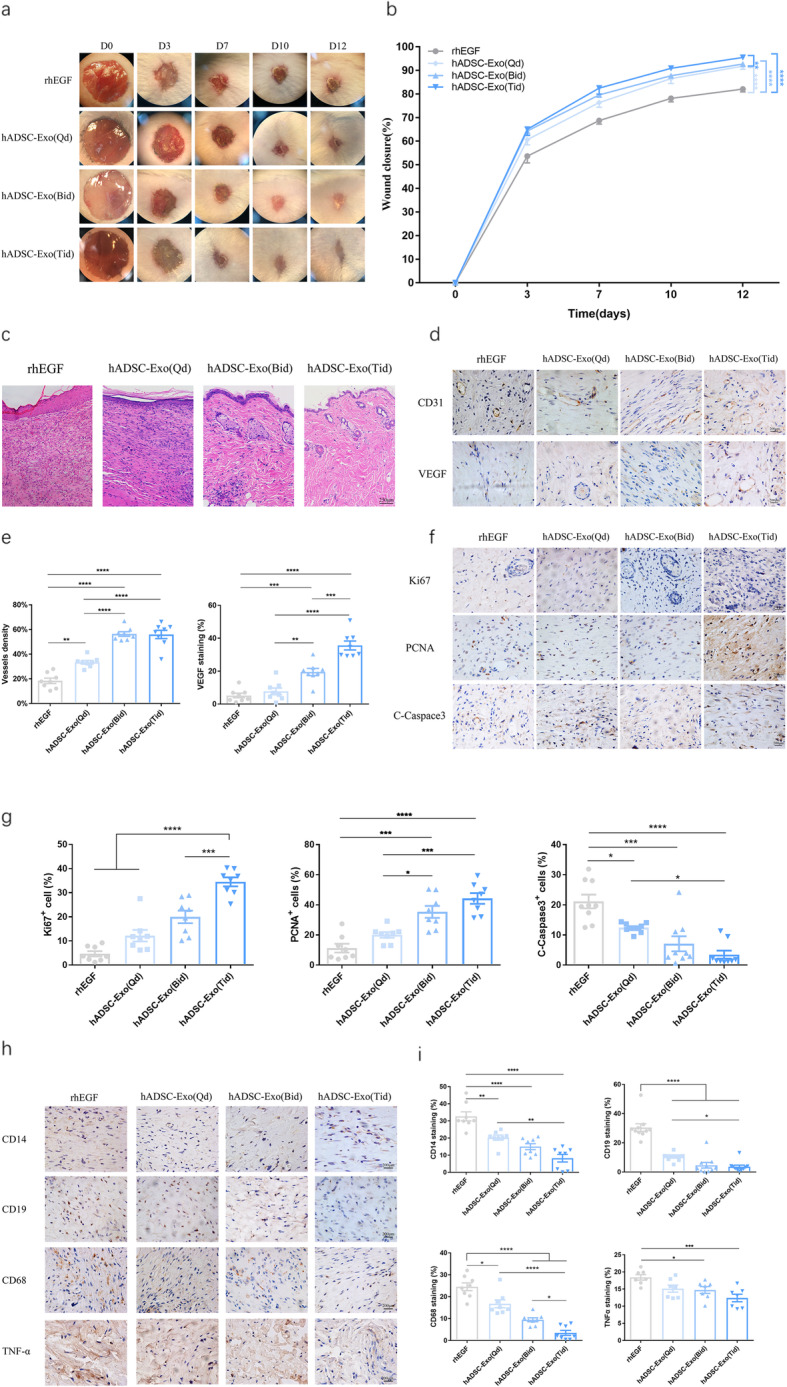
Effect of hADSC-Exo treatment on wound healing in vivo mouse model in a dose-dependent manner. **a** Representative photographs of full-thickness excisional wounds treated with hADSC-Exo (Qd), hADSC-Exo (Bid), and hADSC-Exo (Tid). **b** Quantitative analysis of wound healing in each group. **c** Histological structure of wounded skin in different groups. Scale bar = 250 μm. **d** Representative photographs of CD31 and VEGF immunostaining. Scale bar = 200 μm. **e** Quantitative analysis of the number of mature blood vessels. **f** Representative photographs of Ki67, PCNA, and C-Caspase3 immunostaining. Scale bar = 200 μm. **g** Ki67, PCNA, and C-Caspase3 expression of IHC-positive staining analyzed by mean percentage (±SEM). **h** Representative photographs of CD14, CD19, CD68, MPO, and TNF-alpha immunostaining. Scale bar = 200 μm. **i** Quantification of CD14+, CD19+, CD68+, and TNF-alpha+ IHC stained tissues. The rhEGF was used as a positive control. Results are presented as mean ± standard error of the mean; *n* = 8 for each group. *p < 0.05, **p < 0.01, ***p < 0.001, and ****p < 0.0001 vs vehicle control group

We performed further in vivo investigations to establish whether hADSC-Exo-sm treatment can accelerate the wound closure rate in a dose-dependent manner. To better simulate the local clinical administration of skin wounds, we smeared with three different administration frequencies of hADSC-Exo instead of just increasing the hADSC-Exo dose. The rhEGF was used as a positive control. hADSC-Exo-sm was carried out by three different administration frequencies for up to 12 days of continuous administration: Qd: once a day, Bid: twice a day, and Tid: three times a day. As indicated in Fig. 3a, b, the full-thickness wound mice receiving treatment of hADSC-Exo-sm three times a day exhibited a higher wound closure rate than that treated with other administration at days 3, 7, 10, and 12 post-wounding. Besides, the wounds treated with hADSC-Exo-sm three times a day showed markedly enhanced re-epithelialization at day 12 post-wounding (Fig. 3c). Meanwhile, the greater extent of angiogenesis, the more enhanced proliferation, and the better-suppressed inflammation were reported in the wound sites treated with a three-times-a-day frequency of hADSC-Exo-sm (Fig. 3d–i).

### hADSCs-Exo-sm promoted collagen synthesis, skin barrier repair in vivo

**Fig. 4:**
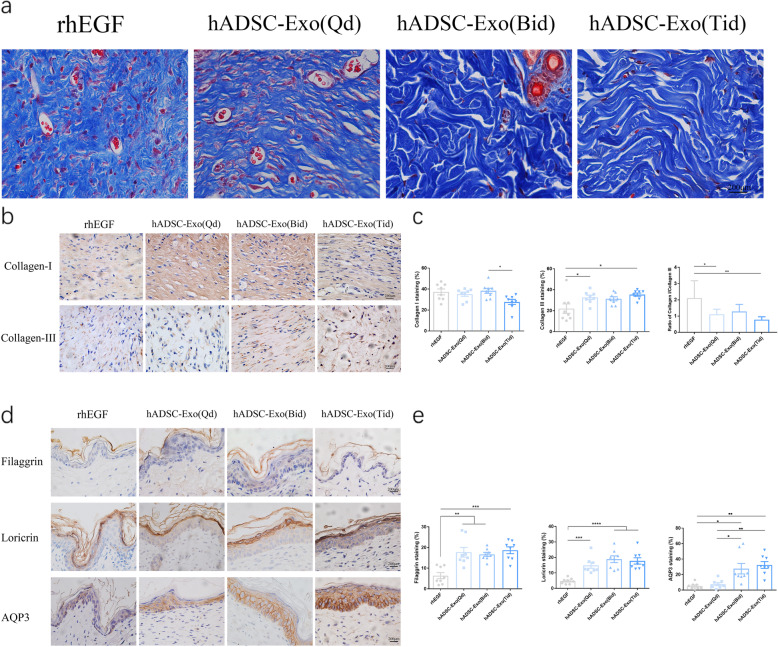
Histological assessment of scar after wound healing in different groups. **a** Masson staining of wounded skin sections in different groups at day 12 post-wounding. Scale bar = 200 μm. **b** The results of immunohistochemical analysis of collagen I and collagen III were same as above. Scale bar = 200 μm. **c** Quantitative analysis of collagen I, collagen III, and the ratio of collagen I/collagen III. **d** Immunohistochemical analyses were performed by using anti-Filaggrin, anti-Loricrin, and anti-AQP3 antibodies in skin wounds. Scale bar = 200 μm. **e** Quantification of Filaggrin, Loricrin, and AQP3 IHC stained tissues. The rhEGF was used as a positive control. Results are presented as mean ± standard error of the mean; *n* = 8 for each group. *p < 0.05, **p < 0.01, ***p < 0.001, and ****p < 0.0001 vs vehicle control group

Further in vivo studies were performed to determine whether hADSC-Exo can affect collagen synthesis ability in the wound site. As indicated in Fig. 4a, higher collagen maturity was reported via Masson staining in wounds treated with ADSC-Exo-sm than the other groups. IHC staining (Fig. 4b, c) of collagen I and collagen III were conducted to visualize the effect of hADSC-Exo-sm on fibroblasts. Similar findings were reported that hADSC-Exo-sm promoted the deposition of collagens. With the increase of hADSC-Exo smearing administration frequency, the wound area showed a greater extent of collagen maturity, and the ratios of collagen I to collagen III were decreased. Scarless healing fetal wounds aggregate more collagen type III relative to scarring adult wounds with a higher proportion of type I collagen deposition [27–30]. These findings precisely demonstrate that hADSC-Exos modulate the deposition and organization of collagen to enhance a scarless pattern.

The epithelium’s outer layer is referred to as the stratum corneum, which accounts for the permeability of the skin barrier and the resilience of the cornified epithelium. Filaggrin contributes to forming relevant components for pH maintenance, moisture, and skin protection against microbial agents. Loricrin is an insoluble protein that forms inter-and intra-protein crosslinks that are highly resistant to proteolysis and stabilize and strengthen the epithelium structure. AQP3 is the predominant aquaporin in the human skin, located in the epidermis’s basal layer [31] and the stratum corneum [32]. These skin barrier proteins are essential to control the epidermis’ selective permeability to build a barrier against the external environment. IHC analysis results showed that hADSC-Exo-sm three times a day improves skin hydration and epidermal permeability barrier function by stimulating the expression of Filaggrin, Loricrin, and AQP3 (Fig. 4d, e).

### Systemic administration facilitate the cutaneous wound healing

**Fig. 5:**
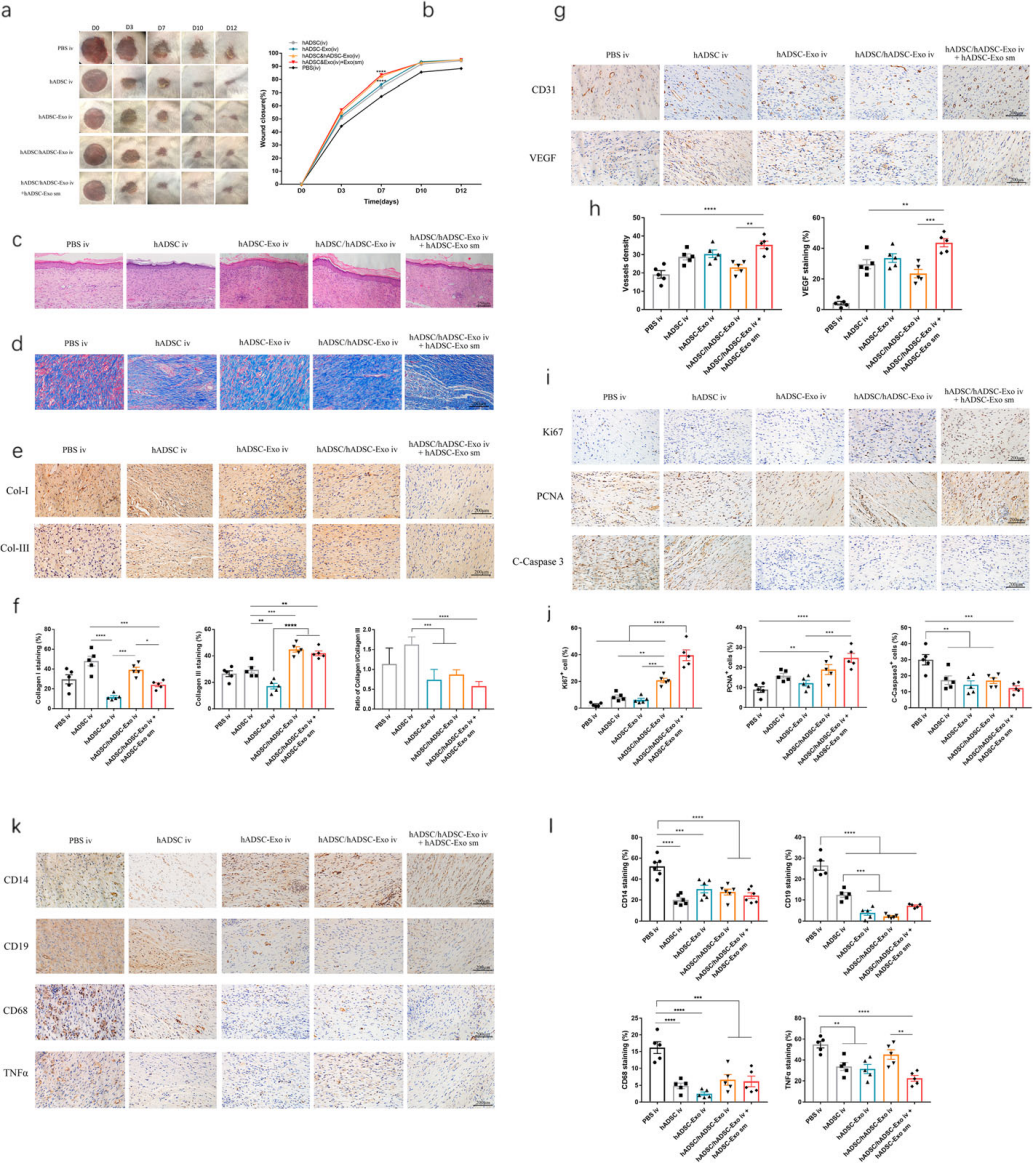
Therapeutic effects of hADSC-Exo in an in vivo mouse model. **a** Representative photographs of full-thickness excision wounds treated with hADSC iv and hADSC-Exo iv with or without hADSC-Exo sm. **b** Quantitative analysis of wound healing in each group. **c** Histological structure of wounded skin in different groups. Scale bar = 250 μm. **d** Masson staining of wounded skin sections in different groups at day 12 post-wounding. Scale bar = 200 μm. **e** The IHC of collagen-I and collagen-III in wounded skin sections on day 12 post-wounding. Scale bar = 200 μm. **f** Quantitative analysis of collagen I, collagen III, and the ratio of collagen I/collagen III. **g** Representative images of immunohistochemical results of CD31 and VEGF. Scale bar = 200 μm. **h** Quantitative analysis of the number of mature blood vessels. Quantitative analysis of the positive cells in the membrane tissues. **i** Representative images of immunohistochemical results of Ki67, PCNA, and C-Caspase3. Scale bar = 200 μm. **j** Quantitative analysis of the positive cells in the skin tissues. **k** Representative photographs of CD14, CD19, CD68, and TNF-alpha immunostaining. Scale bar = 200 μm. **l** Quantification of CD14+, CD19+, CD68+, and TNF-alpha+ IHC stained tissues. Results are presented as mean ± standard error of the mean; *n* = 5 for each group. *p < 0.05, **p < 0.01, ***p < 0.001, and ****p < 0.0001 vs vehicle control group

To investigate the effect of systemic administration for cutaneous wound healing, we performed the intravenous administration with hADSC and hADSC-Exo at 24 h posting-wounding. We found that iv administration of hADSC and hADSC-Exo significantly accelerated wound closure and reduced scar formation (Fig. 5a, b). The wounds treated with iv of hADSC/hADSC-Exo and topical application of hADSC-Exo-sm, compared to other groups, exhibited remarkably enhanced re-epithelialization as indicated in Fig. 5c. Masson staining (Fig. 5d) and IHC staining of Col-I and Col-III (Fig. 5e, f) demonstrated that hADSC/hADSC-Exo iv and hADSC-Exo-sm treated wounds exhibited well-reorganized collagen fibers relative to the other treatment groups post-wounding. The findings illustrated that exosome treatment increased collagen III content in the wound’s bed, and it is organized in a subtle reticular trend. hADSC-Exo sm or hADSC-Exo iv changed the ratio of collagen I to collagen III from a scar-enhancing higher ratio to an anti-scarring lower ratio and decreased the degree of scarring.

In contrast with the other groups, the expression contents of CD31 and VEGF were significantly increased, demonstrating a valid promotion of angiogenesis at the site of wound with hADSC/hADSC-Exo iv and hADSC-Exo-sm treatment (Fig. 5g, h). The numbers of Ki67 and PCNA cells increased in the skin lesions while the number of C-Caspase 3 cells decreased (Fig. 5i, j), indicating that the hADSC/hADSC-Exo iv and hADSC-Exo-sm treatment can promote cell proliferation and inhibit apoptosis. The hADSC/hADSC-Exo iv and hADSC-Exo-sm treatment also remarkably reduced expression of the inflammatory cytokines consisting of CD14, CD19, CD68, and TNF-α in skin lesions of ICR mice (Fig. 5k, l). All the above results revealed that hADSC/hADSC-Exo iv and hADSC-Exo-sm treatment could effectively accelerate the skin wound healing and remodeling the skin structure, which provides reliable research data for future clinical application of hADSC/hADSC-Exo in the cutaneous wound.

## Discussion

Herein, we investigated and compared the therapeutic effects of hADSC and hADSC-Exo on wound healing and cutaneous regeneration. Our findings illustrated for the first time that a combination treatment of hADSC and hADSC-Exo significantly promotes cutaneous wound healing, collagen synthesis, vascularization, and skin barrier repair at wound sites in a mouse full-thickness skin defect model. For topic application, hADSC-Exo smearing improves wound healing more than hADSC. For systemic delivery, combined therapy with the intravenous injection of hADSC-Exo and hADSC offered additional benefit over either therapy alone to enhance cutaneous regeneration.

Studies have opined that the primary mechanism responsible for therapy involving stem cell transplantation is paracrine, with exosomes actively contributing to stem cells’ paracrine properties [33, 34]. Induced pluripotent stem cells (iPSCs), iPSC-derived mesenchymal stem cells (iMSCs), and MSC-derived exosome therapeutic effect in cutaneous wound healing have been reported previously [35–37]. Some preclinical experiments have shown that exosomes from iPSC or iMSCs exert superior functions compared with exosomes derived from MSCs (MSC-Exo) [37]. Although iPSCs possess the self-renewal ability and can differentiate into any types of adult cell, they are not linked to any ethical issues and can serve as a great arsenal of stem cell transplantation therapy; they are possibly tumorigenic [38]. Conversely, the proliferation along with the differentiation abilities of MSCs are limited after numerous passages in culture [39, 40]. Mounting research evidence documents that hADSC is a promising stem cell repertoire for regenerative medicine given their benefits of being acquired conveniently and regeneration ability [41].

Studies on the utility of hADSC-Exo have reported accessibility, as well as promising application in wound healing. In contrast with hADSC-mediated therapy, hADSC-originated exosomes mimic the function of the host cells and could effectively avert the risk of tumor development, immune-induced rejection, limited survival of cells, and loss of function or senescence-triggered genetic instability. Thus, herein, combined therapy with hADSC and hADSC-Exo was first adopted to examine their impacts in cutaneous wound healing in a mouse full-thickness skin defect model.

The typical process of skin regeneration can be summarized into four overlapping phases, namely the inflammation stage, the angiogenesis stage, the proliferation stage consisting of cell proliferation along with reepithelization, and the final remodeling stage. Several studies currently demonstrated the role of MSC-exosomes in the above three stages of wound healing and cutaneous regeneration. Inflammation constitutes the initial response of the four phrases of the typical wounding repair. We found that hADSC and hADSC-Exo can alleviate the inflammatory response resulting from numerous stimuli by downregulating cytokines and chemokines, like tumor necrosis factor (TNF)-α, interleukin (IL)-6, CD14, CD19, and CD68. During the proliferation stage, neoangiogenesis, collagen deposition, re-epithelialization, and wound contraction concur. Neoangiogenesis formation is a pivotal step in wound healing, as well as tissue repair [42]. The results showed that the treatment of hADSC-Exo could increase the expression of angiogenesis-associated molecules consisting of VEGF and CD31. The results also showed that hADSC-Exo promoted wound healing, enhanced re-epithelialization, elevated the expression of Ki67, PCNA, and decreased the expression of C-Caspase 3. The remodeling of the extracellular matrix normally lasts for 2 weeks to more than 1 year. The remodeling stages of wound healing are closely linked to the production of the ECM and its reorganization, which is vital in establishing the scarring extent. The synthesis and degeneration of collagen constitute the key to ECM reconstruction. The narrower scars were observed in wounds treatment with hADSC-Exo(sm) three times a day compared with other groups in our study, and our findings revealed that hADSC-Exo increased the ratios of collagen III to collagen I in the late stage to suppress scar tissue formation, which is one of the prospective mechanisms of reducing scar formation. At last, we investigate the effect of hADSC-Exo on the barrier function recovery of the skin wound, and the results demonstrated that hADSC-Exo increased the expression levels of some skin barrier proteins, like Filaggrin, Loricrin, and AQP3, suggested that hADSC-Exo is beneficial for promoting skin elasticity and barrier integrity. These findings provide strong in vitro research evidence that hADSC-Exo has a prospective clinical utility ability in wound healing and regeneration.

For local skin wounds, hADSC-Exo-sm showed markedly shorten healing time and enhanced re-epithelialization. However, the hADSC-Exo-sm alone is inadequate for the treatment of extensive burns and scalds. So, hADSC-Exo-sm combined therapy with the intravenous injection of hADSC-Exo and hADSC was necessary for clinical extensive burns and scalds. We administered hADSC-Exo to the skin wound of mice via intravenous injection, local smearing, and local injection. The data illustrated that the mice treated with exosomes healed faster than the control mice suggested that Hadsc-Exo enhance cutaneous wound healing. Of note, intravenous injection, and local smearing demonstrated superior wound healing ability relative to local injection, and we attributed this to the loss of exosomes during the local injection. Besides, when directly injecting exosomes into the wound, the wound can be inevitably disturbed, hence disturbing the wound healing process. After gaining entry into blood circulation, exosomes could be mobilized to the injured site and enhance wound healing. However, in addition to these, when intravenous injection of exosomes together with hADSC, a better efficacy was observed in wound healing, which may be attributed to the interaction between hADSC-Exo and hADSC. hADSC-Exo created the local microenvironment for hADSC living in vivo, and meanwhile, the living hADSC would release more exosomes to repair the wound.

## Conclusion

In summary, hADSC can effectively promote skin wound healing while inhibiting scar formation at the wound. As a communication tool between cells, exosomes derived from hADSC can effectively replace hADSC as effective drugs for skin wound repair. Especially for extensive burns, trauma, or ulcers of various etiology, the combined treatment with the intravenous injection of hADSC-Exo and hADSC supplemented with the local application of smearing hADSC-Exo would be one of the best treatment options for clinical patients. Our study provides sufficient evidence and ideas for the clinical treatment of acute or chronic wounds.

## Supplementary Information

**Additional file 1:** **Supplementary material.** The complete WB pictures of CD9 and CD63.

## Data Availability

Data and reagents will be provided upon availability and reasonable request.

## Abbreviations

hADSC: Human adipose-derived mesenchymal stem cells
hADSC-Exo: Human adipose-derived mesenchymal stem cell-derived exosomes
PCNA: Proliferating cell nuclear antigen
VEGF: Vascular endothelial growth factor
TNF-alpha: Transforming growth factor alpha
TEM: Transmission electron microscopy
NTA: Nanosight tracking analysis
PBS: Phosphate-buffered saline
SEM: Scanning electron microscopy
