# Supplementary figures and images for: Combined topical and systemic administration with human adipose-derived mesenchymal stem cells (hADSC) and hADSC-derived exosomes markedly promoted cutaneous wound healing and regeneration
Source: Stem Cell Res Ther. 2021 May 1;12:257. doi: 10.1186/s13287-021-02287-9 (PMC8088044; doi:10.1186/s13287-021-02287-9)

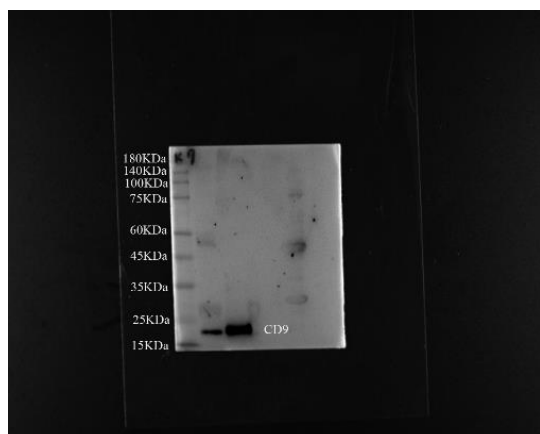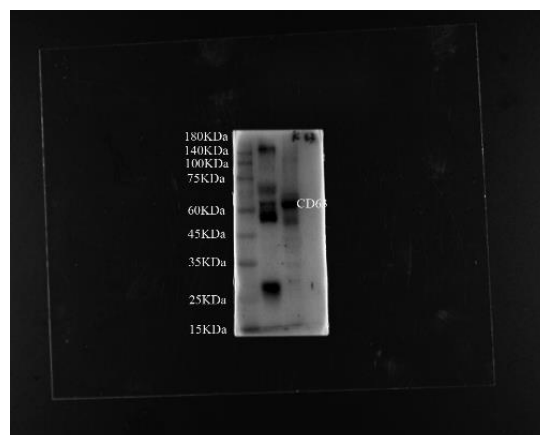

Supplement: Supplementary file 1 — Additional file 1: Supplementary material. The complete WB pictures of CD9 and CD63. [file 13287_2021_2287_MOESM1_ESM.pdf]
